# INPH and parkinsonism: A positive shunt response with a negative tap test

**DOI:** 10.3389/fneur.2023.1150258

**Published:** 2023-03-29

**Authors:** Giulia Giannini, Ignacio Jusue-Torres, Paolo Mantovani, Liliana Mazza, Alessandro Pirina, Nicola Valsecchi, David Milletti, Luca Albini-Riccioli, Sabina Cevoli, Sevil Yasar, Giorgio Palandri

**Affiliations:** ^1^Unit of Neurology, IRCCS Istituto delle Scienze Neurologiche di Bologna, Bologna, Italy; ^2^Department of Biomedical and Neuromotor Sciences (DIBINEM), University of Bologna, Bologna, Italy; ^3^Department of Neurological Surgery, Mayo Clinic, Rochester, MN, United States; ^4^Unit of Neurosurgery, IRCCS Istituto delle Scienze Neurologiche di Bologna, Bologna, Italy; ^5^Dipartimento dell'Integrazione Geriatria, Ospedale Maggiore, AUSL Bologna, Bologna, Italy; ^6^Unit of Rehabilitation Medicine, IRCCS Istituto delle Scienze Neurologiche di Bologna, Bologna, Italy; ^7^Unit of Neuroradiology, IRCCS Istituto delle Scienze Neurologiche di Bologna, Bologna, Italy; ^8^Division of Geriatric Medicine and Gerontology, Johns Hopkins University School of Medicine, Baltimore, MD, United States; ^9^Department of Neurology, Johns Hopkins School of Medicine, Baltimore, MD, United States

**Keywords:** normal pressure hydrocephalus, parkinsonism, shunt, cohort study, tap test

## Abstract

**Introduction:**

The aim of this study was to compare clinical and functional performances of idiopathic normal pressure hydrocephalus (INPH) patients with and without parkinsonism at the initial evaluation, 72 h after the cerebrospinal fluid tap test (CSF TT), and 6 months after ventriculoperitoneal shunt (VPS) surgery.

**Materials and methods:**

This is an observational prospective study on patients with INPH who underwent VPS. Patients were classified into INPH with parkinsonism (INPH-P^+^) and without parkinsonism (INPH-P^−^). We used the time up and go (TUG) test, Tinetti Performance-Oriented Mobility Assessment (POMA) test, INPH grading scale (INHPGS), and modified Rankin scale (mRS) at baseline, 72 h after CSF TT, and 6 months after VPS surgery.

**Results:**

A total of 64 patients with probable INPH were included, 12 patients with INPH-P^+^ and 52 controls with INPH-P^−^. Patients with INPH showed significant improvement in all clinical and neurological parameters after VPS including TUG, Tinetti POMA, INPHGS, and mRS (*p* < 0.001) with the exception of mRS where there was no significant change 72 h after CSF TT compared to baseline for patients with INPH (*p* = 0.182). Patients with INPH-P^+^ performed significantly worse than patients with INPH-P^−^ on Tinetti POMA and mRS at baseline, at 72 h post-CSF TT, and at 6 months post-VPS with INPHGS being worst at 72 h post-CSF TT. There was no difference between patients with INPH-P^+^ and patients with INPH-P^−^ for TUG at baseline (*p* = 0.270), at 72 h post-CSF TT (*p* = 0.487), and at 6 months post-VPS (*p* = 0.182). Patients with INPH-P^+^ did not show any change in any of the parameters at 72 h post-CSF TT compared to baseline; however, there was a trend toward improvement on TUG (*p* = 0.058), Tinetti gait (*p* = 0.062), and Tinetti total (*p* = 0.067). INPH-P^+^ significantly improved in all parameters 6 months post-VPS compared to baseline except for mRS (*p* = 0.124). Patients with INPH-P^−^ significantly improved in all parameters at 72 h post-CSF TT and at 6 months post-VPS compared to baseline, respectively, except on mRS 72 h after CSF TT (*p* = 0.299).

**Conclusion:**

Patients with INPH and parkinsonism overall do worse than patients without parkinsonism. An unsatisfying response to the CSF tap test in INPH patients with parkinsonism should not be used as an exclusion criterion from VPS surgery since patients with and without parkinsonism showed significant improvement post-VPS.

## Introduction

A clinical evaluation of patients with suspected idiopathic normal pressure hydrocephalus (INPH) is a challenging endeavor. Neurological comorbidities may overlap, resulting in a confusing clinical picture and a complicated indication for shunt surgery (1, 2). In particular, parkinsonism should be carefully evaluated as it may represent either an additional symptom or a feature of a neurodegenerative disease mimicking a clinical picture of INPH or the manifestation of a second co-existing pathology. Parkinsonism is defined by bradykinesia associated with at least one other extrapyramidal sign (rigidity, resting tremor, and/or postural instability) (3) and occurs in different neurodegenerative conditions, including Parkinson's disease (PD), corticobasal degeneration, Lewy body dementia, progressive supranuclear palsy, multiple system atrophy, and frontotemporal dementia.

The prevalence of parkinsonism in suspected INPH is reported with high variability (20–86%) (4, 5) among categories of INPH-mimics (possible and probable INPH) according to current diagnostic criteria (6, 7). For these patients, clinical and operative approaches have evolved over the years, from an initial exclusion from shunt indication to a more recent inclusion on the basis of objective improvement of parkinsonian symptoms after cerebrospinal fluid (CSF) diversion.

Improvement of parkinsonian symptoms after the cerebrospinal fluid tap test (CSF TT) and shunt surgery has been reported (8–10); however, clinical and neurological performance comparison between with and without parkinsonism has been less investigated.

The aim of this study was to compare clinical and functional performances of INPH patients (7) with (INPH-P^+^) and without (INPH-P^−^) parkinsonism at baseline evaluation, 72 h after the tap test (TT), and 6 months after ventriculoperitoneal shunt (VPS) surgery.

## Materials and methods

### Patients' selection

This is an observational prospective study on patients with INPH belonging to the Bologna PRO-Hydro cohort (11). The Bologna PRO-Hydro study prospectively recruited patients with suspected INPH and underwent MRI protocol and multidisciplinary evaluation, as described earlier (12). Patients with suggestive clinical symptoms and neuroradiological signs of INPH were enrolled in the inpatient program, including thorough clinical evaluation and standardized CSF TT (described in the Clinical Evaluation Section). The diagnosis was assigned after reviewing all pre-TT clinical data and neuropsychological information, blood/CSF tests, and comparisons between pre- and post-TT during a consensus case conference involving the multidisciplinary team (11). On the basis of clinical history, physical examination, CSF biomarker profile, and the severity of comorbidities, the multidisciplinary team established eligible patients for the VPS surgery (a standardized procedure with the implant of CODMAN^®^ HAKIM^®^ programmable valves with the SIPHONGUARD^®^ system).

Given the high rate of false negatives and the high complexity of these kinds of patients, the multidisciplinary team established eligible patients without defining “*a priori*” cutoff response at the TT in the considered variables (motor performances including gait analysis, postural stability, urinary symptoms, and appropriate scales), and a “negative response” in one or multiple items was not sufficient to exclude eligibility for the VPS surgery.

Clinical evaluation and protocol MRI were repeated 6 months after surgery.

For the purpose of this study, we included patients with INPH belonging to the Bologna PRO-Hydro cohort (11), diagnosed as probable INPH according to consensus guideline criteria (7), and undergoing VPS surgery. Patients with partial clinical assessment or lacking appropriate neuroimaging studies were excluded.

We defined INPH-P^+^ as probable INPH with bradykinesia and at least one other extrapyramidal sign (tremor or rigidity, or both), as defined by the Movement Disorder Society (MDS) Clinical Diagnostic Criteria for PD (13). A subjective clinical benefit after levodopa intake and/or the objective levodopa response was further required for being included in the INPH-P^+^ group.

The absence of the abovementioned criteria was required for the classification of patients with INPH without parkinsonism (INPH-P^−^).

The study was conducted in agreement with the principles of good clinical practice. The study protocol was approved by the Local Ethics Committee of the local health service of Bologna, Italy (Cod. CE: 14131). All patients gave their written informed consent to study participation.

### Clinical evaluation

The clinical evaluation of INPH cohort patients was performed at three different times: baseline, 72 h after CSF TT, and 6 months after VPS. The severity of INPH symptoms was assessed with the INPH grading scale (INPHGS) (0–12 points) (14). The Tinetti Performance-Oriented Mobility Assessment (POMA) (15) test was used to evaluate gait (0–12 points) and balance (0–16 points) (Tinetti POMA total score (0–28 points). Timed up and go (TUG) test measured the time the patient took to rise from a chair (in seconds), walk 3 m, turn around, walk back to the chair, and sit down (16). Overall disability and dependence in daily activities were assessed using a modified Rankin score (mRS) (17).

### Statistical analysis

We compared INPH-P^+^ and INPH-P^−^ using the unpaired Wilcoxon rank sum test with continuity correction for continuous variables and Fisher's exact for categorical variables. We used pairwise analysis to compare the repeated measures obtained during the earlier described three different timepoints (admission (baseline), 72 h after CSF TT, and 6 months after VPS) for INPH-P^+^ and INPH-P^−^. As previously mentioned, the multidisciplinary team did not define an “a priori” cutoff response at the TT. In this study, we compared the repeated measures evaluating differences in the continuous values of the considered clinical scales in the two groups (INPH-P^+^ and INPH-P^−^), without a defined categorical variable of TT response. We used the paired Wilcoxon rank sum test with continuity correction for the analysis comparing the different timepoints. The nominal significance level was set at *p* = 0.05. Statistical analysis was performed using R statistic software version 4.2.1 (18).

## Results

### Baseline demographic characteristics of all patients with INPH

From May 2015 to November 2019, 104 patients with suspected INPH were referred to our Institute and were studied by the Pro-Hydro team (11): 64 patients fulfilled the criteria for probable INPH, received high-volume CSF TT during inpatient evaluation, and, subsequently, underwent VPS placement.

There were 27 female patients (42%) and 37 male patients (58%), the median age at evaluation was 75 years (min 65, max 84), and the median BMI was 26.50 Kg/m (2) (min 17.97, max 34.85).

Of the 64 patients included in this study, 12 were classified as INPH-P^+^ and 52 as INPH-P^−^ (Table 1). A total of 52 patients completed 6 months of follow-up evaluation after surgery.

**Table 1:** Baseline characteristics of the 64 INPH patients comparing INPH patients with and without parkinsonism.

**Patient characteristics**	**INPH-P^−^(*N* = 52)**	**INPH-P^+^ (*n* = 12)**	***p*-value**
Age	75 (65–84)	75 (68–79)	0.564
Female	23 (44%)	4 (33%)	0.537
BMI	26 (18–35)	28 (24–35)	0.174
Charlson CMI	0 (0–4)	1 (0–3)	0.364
**Metrics at presentation**			
TUG (sec)	19.36 (10.63–38.17)	18.29 (12.77–92.5)	0.270
Tinetti gait	**8 (1–12)**	**3.5 (1–10)**	**0.002**
Tinetti balance	**13 (3–16)**	**10 (1–15)**	**<0.001**
Tinetti total	**20 (6–28)**	**13.5 (2–23)**	**0.011**
INPHGS	6 (1–12)	6.5 (2–9)	0.170
Modified rankin scale	**2 (1–5)**	**3 (1–4)**	**0.019**

Values are expressed as median (minimum–maximum). CMI, comorbidity index; INPHGS, INPH grading scale; NPH-P^−^, NPH patient without parkinsonism; NPH-P^+^, NPH patient with parkinsonism; TUG, timed up and go test. Statistically significant *p* values are denoted in bold.

At the baseline evaluation, the median TUG for all patients was 18.99 s (min 10.63, max 92.5 s). The Tinetti POMA total median score at initial baseline evaluation was 20 (min 2, max 28), with a median balance score of 13 (min 1, max 16) and a median gait score of 7 (min 1, max 12). The median INPH grading scale at presentation was 6 (min 1, max 12). The median modified Rankin score (mRS) at presentation was 2 (min 1, max 5).

### Gait testing and scales 72 h post-CSF TT in all patients with INPH

At 72 h post-CSF TT, median TUG showed significant improvement in the total sample by decreasing to 16.57 seconds (min 8.80, max 48.80) (*p* < 0.001). Tinetti POMA total median score significantly improved by increasing to 23 (min 9, max 28) (*p* < 0.001), and this improvement was present in both gait and balance median scores 9 (*p* < 0.001) and 14 (*p* < 0.001), respectively. INPHGS significantly improved by decreasing to 4 (min 1, max 9) (*p* < 0.001), while mRS did not change significantly (median of 2; min 0, max 4) (*p* = 0.182).

### Gait testing and scales 6 months after VPS in all patients with INPH

At 6 months after VPS, participants showed significant improvement in every parameter (*p* < 0.001). The median TUG score significantly improved by decreasing to 14.3 (min 9.63, max 42.1). The Tinetti POMA total median score significantly improved by increasing to 24.5 (min 15, max 28), and this improvement was present in both gait and balance median scores 10 (min 2, max 12) and 15 (min 9, max 16), respectively. The median INPHGS score significantly improved by decreasing to 4 (min 0, max 11) while the median MRS score also significantly improved by decreasing to 1 (min 0, max 5).

### Gait testing and scales 72 h post-CSF TT and 6 months after VPS in patients with INPH-P^+^ compared to INPH-P^−^

Among the 64 patients that fulfilled the inclusion criteria, 12 were classified as INPH-P^+^ and 52 as INPH-P^−^. Bradykinesia was present in all 12 cases of INPH-P^+^, where 10 patients presented rigidity, seven patients had tremor, and five patients had both tremor and rigidity. Overall, there were no statistically significant differences between INPH-P^+^ and INPH-P^−^ for age, sex, BMI, and Charlson's Comorbidity Index (Table 1).

**Figure 1:**
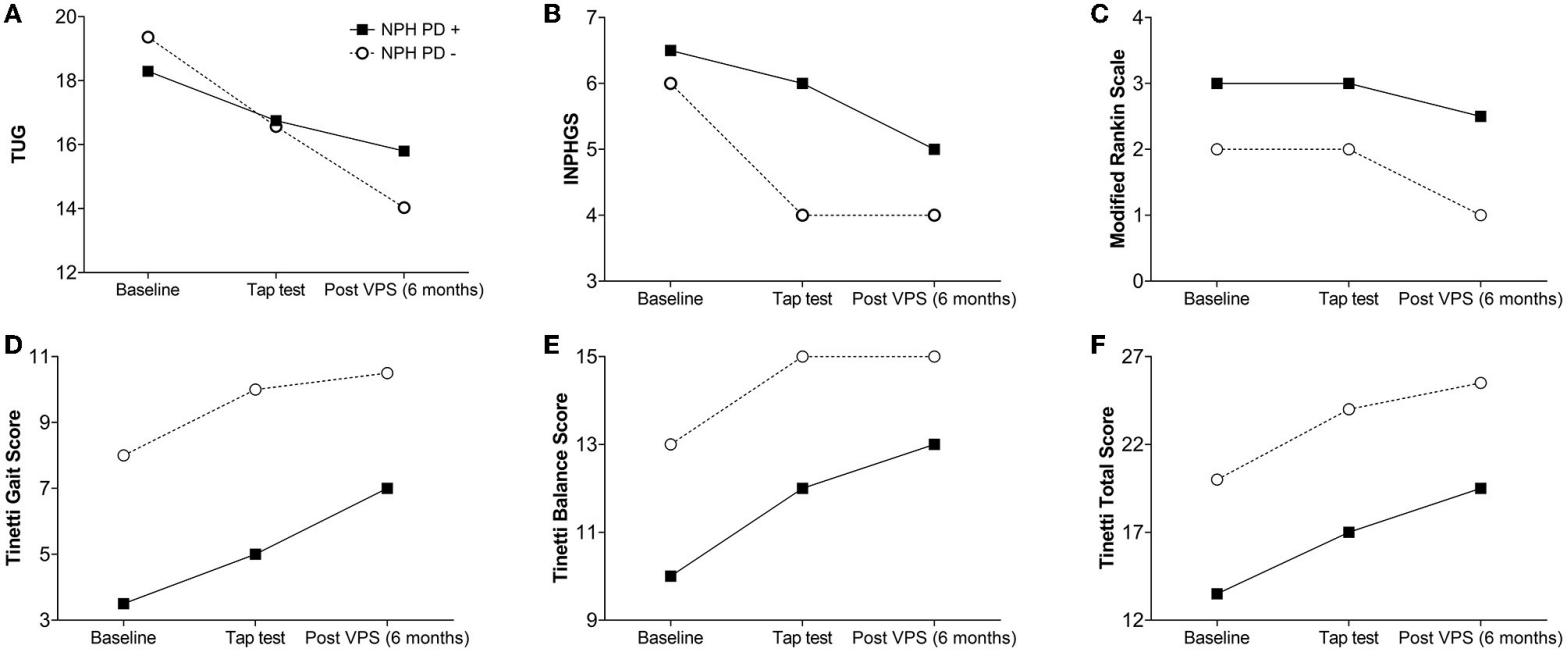
Spaghetti plot comparing the changes overtime for INPH patients with (INPH-P^+^) and without parkinsonism (INPH-P^−^) at baseline, 72 h after CSF TT, and 6 months after shunt surgery. **(A)** TUG, **(B)** INPHGS, **(C)** modified Rankin scale, **(D)** Tinetti Gait Score, **(E)** Tinetti Balance Score, and **(F)** Tinetti Total Score.

At baseline, the TUG test and INPH grading scale showed no statistically significant differences when comparing the two groups (*p* = 0.270 and *p* = 0.170, respectively) (Tables 1, 2 and Figures 1A, B). Patients with INPH-P^+^ had significantly worst median values on Tinetti gait (*p* < 0.002), Tinetti balance (*p* < 0.001), Tinetti total (*p* < 0.011), and MRS (0.019) scores at baseline when compared to INPH-P^−^ (Tables 1, 2 and Figures 1C, D).

**Table 2:** Comparison of INPH patients without parkinsonism (NPH-P^−^) vs. INPH patients with parkinsonism (NPH-P^+^) at presentation/baseline, 72 h after CSF TT, and at 6 months after VPS surgery.

	**Presentation/baseline**			**72 h after CSF TT**			**Post-VPS (6 months)**		
	**INPH-P**^−^**(*****N*** = **52)**	**INPH-P**^+^ **(*****n*** = **12)**	***p*** **-value** ^*^	**INPH-P**^−^**(*****N*** = **52)**	**INPH-P**^+^ **(*****n*** = **12)**	***p*** **-value** ^*^	**INPH-P**^−^**(*****N*** = **52)**	**INPH-P**^+^ **(*****n*** = **12)**	***p*** **-value** ^*^
TUG (sec)	19.36 (10.63–38.17)	18.29 (12.77–92.5)	0.270	16.56 (8.80–31.90)	16.75 (12.77–48.80)	0.487	14.03 (9.63–42.10)	15.80 (11.40–24.98)	0.128
Tinetti gait	8 (1–12)	3.5 (1–10)	**0.002**	10 (4–12)	5 (3–9)	**<0.001**	10.5 (2–12)	7 (5–12)	**0.016**
Tinetti balance	13 (3–16)	10 (1–15)	**<0.001**	15 (7–17)	12 (6–15)	**0.002**	15 (11–16)	13 (9–15)	**0.0014**
Tinetti total	20 (6–28)	13.5 (2–23)	**0.011**	24 (11–28)	17 (9–24)	**<0.001**	25.5 (15–28)	19.5 (15–25)	**0.002**
INPHGS	6 (1–12)	6.5 (2–9)	0.170	4 (1–9)	6 (2–9)	**0.014**	4 (0–11)	5 (3–7)	0.106
Modified rankin scale	2 (1–5)	3 (1–4)	**0.0189**	2 (0–4)	3 (1–4)	**0.012**	1 (0–5)	2.5 (1–4)	**0.002**

Values are expressed as median (minimum–maximum). INPHGS, INPH grading scale; NPH-P^−^, NPH patient without parkinsonism; NPH-P^+^, NPH patient with parkinsonism; TUG, timed up and go test. ^*^Unpaired Wilcoxon rank sum test with continuity correction. Statistically significant *p* values are denoted in bold.

At 72 h after CSF TT, INPH-P^+^ performed worse than patients with INPH-P^−^ in the majority of parameters [Tinetti gait score (*p* < 0.001), Tinetti balance score (*p* = 0.002), Tinetti total score (*p* < 0.001), INPHGS (*p* = 0.014), and MRS (*p* = 0.012)]. The TUG time did not show a difference between groups at this evaluation (*p* = 0.487) (Table 2, Figure 1).

At 6 months after VPS surgery, in a similar fashion than at baseline, there were no statistically significant differences for both TUG and INPHGS when comparing INPH-P^+^ and INPH-P^−^ (*p* = 0.128 and *p* = 0.106, respectively) (Table 2, Figure 1). INPH-P^+^ patients showed significantly worst median values for Tinetti gait (*p* = 0.016), Tinetti balance (*p* = 0.0014), Tinetti total (*p* = 0.002), and mRS (*p* = 0.002) at 6 months after VPS surgery when compared with controls INPH-P^−^.

### Gait testing and scales differences between baseline vs. at 72 h post-CSF TT

When comparing the median parameters of patients with INPH-P^+^ at baseline vs. at 72 h after CSF TT, there were no statistically significant differences in any of the parameters (Table 3 and Figure 1). However, there were trends toward significant improvement in measures of TUG (*p* = 0.058), Tinetti gait (*p* = 0.062), and Tinetti total (*p* = 0.067).

**Table 3:** Comparison of differences between parameters at presentation/ baseline vs. at 72 h after the tap test and at presentation/baseline vs. at 6 months post-VPS for NPH-P^+^ and NPH-P^−^.

	Δ **baseline-CSF TT**^**#**^				Δ **baseline-VPS**^**#**^			
	**INPH-P**^−^**(*****N*** = **52)**		**INPH-P**^+^ **(*****n*** = **12)**		**INPH-P**^−^**(*****N*** = **52)**		**INPH-P**^+^ **(*****n*** = **12)**	
	Δ **(95% conf interval)**	***p*** **-value** ^#^	Δ **(95% conf interval)**	***p*** **-value** ^#^	Δ **(95% conf interval)**	***p*** **-value** ^#^	Δ **(95% conf interval)**	***p*** **-value** ^#^
TUG (sec)	2.79 (1.57–4.25)	**<0.001**	4.75 (−0.68 to 38.06)	**0.058**	4.45 (1.81–6.82)	***p*** **<0.001**	13.75 (1.25–52.21)	**0.034**
Tinetti gait	−2.50 (−3.00 to −2.00)	**<0.001**	−1.50 (−4.00 to 0.00)	**0.062**	−2.50 (−4.00 to −1.50)	***p*** **<0.001**	−4.0 (−7.0 to −2.00)	**0.008**
Tinetti balance	−2.00 (−3.00 to −1.50)	**<0.001**	−1.50 (−3.50 to 1.00)	0.211	−2.50 (−3.00 to −1.50)	***p*** **<0.001**	−3.5 (−6.5 to −0.50)	**0.032**
Tinetti total	−4.0 (−5.00 to −3.00)	**<0.001**	−2.00 (−5.00 to 0.50)	**0.067**	−5.00 (−6.00 to −3.00)	***p*** **<0.001**	−10.00 (−15.50 to −5.00)	**0.014**
INPHGS	1.50 (1.50–2.00)	**<0.001**	2 (1.00–2.99)	0.181	2.5 (1.50–3.00)	***p*** **<0.001**	2.00 (1.50–2.50)	**0.007**
Modified rankin scale	1.00 (0.00–1.00)	0.299	1 (NA–NA)	1	1.00 (1.00–1.50)	***p*** **<0.001**	1.00 (0–2.00)	0.124

Δ, Delta or difference, INPHGS, INPH grading scale; NA, not applicable (cannot compute confidence interval when all observations are tied); NPH-P^−^, NPH patient without parkinsonism; NPH-P^+^, NPH patient with parkinsonism; TUG, timed up and go test. ^#^Paired Wilcoxon rank sum test with continuity correction. Statistically significant *p* values are denoted in bold.

When comparing median parameters of patients with INPH-P^−^ at baseline vs. at 72 h after CSF TT, every single parameter resulted in significant improvement (*p* < 0.0001) with the exception of mRS (*p* = 0.299) (Table 3 and Figure 1).

### Gait testing and scales differences between baseline vs. at 6 months post-VPS

When comparing median parameters of patients with INPH-P^+^ at baseline vs. at 6 months post-VPS, every single variable showed statistically significant improvement except for mRS (*p* = 0.124) (Table 3 and Figure 1).

For INPH-P^−^, when comparing median values at baseline vs. at 6 months post-VPS, every single variable showed statistically significant improvement (*p* < 0.0001) (Table 3 and Figure 1).

## Discussion

This study shows that after rigorous and thorough multidisciplinary evaluation, overall patients with INPH showed significant improvement for all the clinical and neurological parameters after VPS in TUG, Tinetti POMA (gait, balance, and total), INPHGS, and mRS. mRS was the single parameter that did not show significant change 72 h after CSF TT when compared to baseline for all patients with INPH.

Overall, respect to INPH-P^−^, INPH-P^+^ patients showed worse performances in the majority of variables at baseline, 72 h post CSF TT and 6 months after VPS. However, despite this group did not show a significant response after CSF TT, a significant improvement was observed 6 months after VPS. This finding could positively impact the clinical practice as an unsatisfying response to CSF TT in patients with INPH with parkinsonism should not be used as an exclusion criterion from VPS surgery.

In particular, INPH-P^+^ showed significantly worse parameters when compared to INPH-P^−^ for Tinetti POMA (gait, balance, and total) and mRS at baseline, at 72 h after the tap test, and at 6 months post-VPS with INPHGS worst at 72 h post-CSF TT. There were no differences between INPH-P^+^ and INPH-P^−^ patients in tests of TUG at baseline, at 72 h post-CSF TT, and at 6 months post-VPS.

At 72 h post-CSF TT, INPH-P^+^ patients showed no significant change in any of the parameters when compared to baseline, showing trends toward improvement in tests of TUG, Tinetti gait, and Tinetti total. However, at 6 months after VPS, the INPH-P^+^ group showed significant improvement in all parameters compared to baseline except for mRS.

On the contrary, INPH-P^−^ patients showed significant improvement in all parameters 72 h post-CSF TT and at 6 months post-VPS compared to baseline, respectively (except for mRS 72 h after CSf TT).

These results suggest that INPH patients with parkinsonism (INPH-P^+^) had a peculiar clinical profile in all three observation times. At baseline, this group showed greater gait impairment, worst imbalance, and more severe overall disability compared to patients without parkinsonism. This may not be surprising, as the influence of comorbidities such as parkinsonism is reported to worsen the status of patients with INPH (19). However, in our study, there were no statistical differences when comparing the Charlson comorbidity index between INPH-P^+^ and INPH-P^−^.

Our findings are consistent with the data of our recent study conducted on a larger sample (20), founding that 20.5% of INPH patients showed α-synuclein seeding activity in the CSF and that this subgroup of patients had a higher score on axial and upper limb rigidity and presented a more significant gait impairment characterized by petit-pas gait, start hesitation, and reduced gait speed.

Another finding of this study was the non-significant response to CSF TT in INPH patients with parkinsonism: none of the considered clinical and neurological variables reached a significant improvement 72 h after CSF withdrawal. A recent study by Morel et al. (12) documented a similar result 24 h after CSF TT in 12 INPH patients with parkinsonian gait. INPH patients with parkinsonism showed a significant clinical improvement after shunt surgery in almost all considered variables except for mRS although patients without parkinsonism generally gained better postoperative scores. These findings partially confirm the results of Akiguchi et al. (21) and, more recently, of Mostile et al. (22) and Giannini et al. (20). A possible explanation of the discrepancy observed between the CSF TT and VPS response could be both time- and quantity-dependent, as the clinical improvement in patients with parkinsonism may not be relevant after a single CSF withdrawal, while it becomes clinically appreciable after chronic CSF diversion, thus reflecting the well-known limits of this diagnostic test and its relevance to the VPS response (23). On the contrary, in the current study, looking both at the amount of change for Tinetti (gait, balance, and total) and mRS had similar behavior for both INPH-P^+^ and INPH-P^−^. Statistical significance was likely not reached because of the small sample size and therefore small power. Therefore, a negative response or only a slight response to CSF withdrawal should not be a reason to exclude patients from VPS surgery. There is growing evidence that subjective improvements noticed by patients and caregivers can better identify patients who will experience significant clinical improvement post-shunt (24, 25). This could be explained by the fact that most of our tests are performed in a standardized environment that does not represent the real-world uneven challenges for gait and balance. In addition, the baseline is different for every individual and some of our tests fail to detect those changes that make a difference in our patients' daily life.

It appears also necessary to understand which scales and tools are more appropriate to suspect and discover parkinsonism in INPH patients, which in our case series was present in 19% (12/64). Among those included in this study, Tinetti POMA appeared to be discriminant in defining the profiles of the groups, as parkinsonism in INPH seemed to affect gait more severely, directly contributing to a higher risk of fall. Similarly, mRS, which is widely used in clinical practice and outcome evaluation to assess the care need of a neurological patient, well-defines INPH patients with parkinsonism, who seem to have a higher overall disability even after shunt surgery. Conversely, other scales and tools, although widely used in literature for INPH patients, were not able to detect the effect of parkinsonism as the former. In fact, the TUG test, which measures the time to perform a compound motor task, and INPHGS, which defines the severity of INPH disease based on the affection on cognitive, urinary, and gait domains, showed comparable results in the two considered groups in both baselines and after VPS observations.

### Limitations

This study has some limitations including those associated with being a single center with a small sample size, which may create biases associated with patient and treatment selection. Further multicentric studies with a more heterogeneous population, bigger sample, and power are necessary to increase generalizability and to develop and validate better tools to identify patients with hydrocephalus and parkinsonism.

## Conclusion

Patients with INPH and parkinsonism do overall worse than INPH patients without parkinsonism. An unsatisfying response to CSF TT in INPH patients with parkinsonism should not be used as an exclusion criterion from VPS surgery since patients with and without parkinsonism showed significant clinical and neurological improvement post-VPS.

## Data availability statement

The raw data supporting the conclusions of this article will be made available by the authors, without undue reservation.

## Ethics statement

The studies involving human participants were reviewed and approved by Local Ethics Committee of the local health service of Bologna, Italy (Cod. CE: 14131). The patients/participants provided their written informed consent to participate in this study.

## Author contributions

GG, GP, and PM contributed to conception and design of the study and wrote the first draft of the manuscript. PM organized the database. IJ-T performed the statistical analysis. DM, NV, LA-R, ML, SC, SY, and AP wrote sections of the manuscript. All authors contributed to manuscript revision, read, and approved the submitted version.
